# Novel method for reduction of virus load in blood plasma by sonication

**DOI:** 10.1186/s40001-020-00410-9

**Published:** 2020-04-07

**Authors:** D. Pförringer, K. F. Braun, H. Mühlhofer, J. Schneider, A. Stemberger, E. Seifried, E. Pohlscheidt, M. Seidel, G. Edenharter, D. Duscher, R. Burgkart, A. Obermeier

**Affiliations:** 1 Klinikum rechts der Isar der Technischen Universität München, Klinik und Poliklinik für Unfallchirurgie, Ismaninger Str. 22, 81675 Munich, Germany; 2 Charite, Universitätsmedizin Berlin, Unfallchirurgie, Zentrum für Muskuloskeletale Chirurgie, Berlin, Germany; 3 Klinikum rechts der Isar der Technischen Universität München, Klinik für Orthopädie und Sportorthopädie, Ismaninger Str. 22, 81675 Munich, Germany; 4 II. Medizinische Klinik und Poliklinik, Klinikum rechts der Isar der Technischen Universität München, Ismaninger Str. 22, 81675 Munich, Germany; 5 DRK-Blutspendedienst, Institut für Transfusionsmedizin und Immunhämatologie, Sandhofstrasse 1, 60528 Frankfurt, Germany; 6 Institut für Wasserchemie & Chemische Balneologie, Lehrstuhl für Analytische Chemie und Wasserchemie, Technische Universität München, Marchioninistr. 17, 81377 Munich, Germany; 7 Klinikum rechts der Isar der Technischen Universität München, Klinik für Anästhesie, Munich, Germany; 8 Klinikum rechts der Isar der Technischen Universität München, Klinik für Plastische Chirurgie, Ismaninger Str. 22, 81675 Munich, Germany

**Keywords:** Virus, Ultrasound, Sonication, Inactivation, Shielding gas, Cavitation, Coagulation proteins

## Abstract

**Background:**

Aim of the present study is the evaluation of ultrasound as a physical method for virus inactivation in human plasma products prior to transfusion. Our study is focused on achieving a high level of virus inactivation simultaneously leaving blood products unaltered, measured by the level of degradation of coagulation factors, especially in third world countries where virus contamination of blood products poses a major problem. Virus inactivation plays an important role, especially in the light of newly discovered or unknown viruses, which cannot be safely excluded via prior testing.

**Methods:**

Taking into account the necessary protection of the relevant coagulation activity for plasma, the basis for a sterile virus inactivation under shielding gas insufflation was developed for future practical use. Influence of frequency and power density in the range of soft and hard cavitation on the inactivation of transfusion-relevant model viruses for Hepatitis-(BVDV = bovine diarrhea virus), for Herpes-(SFV = Semliki Forest virus, PRV = pseudorabies virus) and Parvovirus B19 (PPV = porcine parvovirus) were examined. Coagulation activity was examined via standard time parameters to minimize reduction of functionality of coagulation proteins. A fragmentation of coagulation proteins via ultrasound was ruled out via gel electrophoresis. The resulting virus titer was examined using end point titration.

**Results:**

Through CO_2_ shielding gas insufflation—to avoid radical emergence effects—the coagulation activity was less affected and the time window for virus inactivation substantially widened. In case of the non-lipidated model virus (AdV-luc = luciferase expressing adenoviral vector), the complete destruction of the virus capsid through hard cavitation was proven via scanning electron microscopy (SEM). This can be traced back to microjets and shockwaves occurring in hard cavitation. The degree of inactivation seems to depend on size and compactness of the type of viruses. Using our pre-tested and subsequently chosen process parameters with the exception of the small PPV, all model viruses were successfully inactivated and reduced by up to log 3 factor. For a broad clinical usage, protection of the coagulation activities may require further optimization.

**Conclusions:**

Building upon the information gained, an optimum inactivation can be reached via raising of power density up to 1200 W and simultaneous lowering of frequency down to 27 kHz. In addition, the combination of the two physical methods UV treatment and ultrasound may yield optimum results without the need of substance removal after the procedure.

## Introduction

Since the introduction of blood transfusion security levels of the blood transfer process between individual humans for medical purposes have been a key issue, periodically aggravated by current virus circulations in society. Initially the topic had become a major concern after the discovery of HIV. The current security concept includes careful donor recruiting under elimination of high-risk groups, testing for presence or absence of virus in donor blood and utilization of newly developed measures for virus inactivation.

In Germany, all blood and plasma products are regarded as pharmaceuticals and thus are subject to pharmaceutical legislation. In 1998 in Germany, a new law on blood transfusion was passed and amended in 2007 and 2017 [1]. Human blood is a valuable resource and raw material for vital components such as: erythrocyte concentrate = packed red blood cells, platelet concentrate, fresh (frozen) plasma (FFP). FFP itself poses the raw material for several components of plasma. Plasma derivatives are produced from plasma pools, consisting of plasma of thousands of donors. This method raises the risk of a potential infection in comparison to a singular plasma donation, as a pool can be contaminated by one single infectious donation. This justifies the intense efforts on behalf of the plasma processing industry to find methods for inactivation of at least all known virus types.

Blood and blood component transfusions can cause transfer of parasites, bacteria and viruses resulting in serious infections [2]. Since 1999, examination for transfusion-relevant virus HCV and since 2004 for HIV via nucleic acid amplification test (NAT) have become mandatory. This leaves a minimum residual risk due to limitations in sensitivity and specificity of analysis methods as well as the pooling method of NAT [3].

Various methods for virus depletion and inactivation are available [4] as outlined. In addition chemical methods for virus inactivation in plasma from pools and in singular plasma are available [5]. Most of these mechanisms additionally damage proteins and reduce activity of coagulation factors.

The solvent detergent method poses the best known and most common, feasible method for lipidated viruses primarily for use on plasma pools. Methylene blue inactivation is not yet authorized in Germany, but is employed in other EU states. It exerts its inactivation via interaction of methylene blue with the genome of the virus and subsequent radiation with sodium light. This generally works for all viruses, but cannot inactivate some non-lipidated viruses such as, e.g., PPV. Inactivation methods using psoralenes (furocumarine), gene toxic substances, are currently in development [6], while the Intercept Blood™ system, using Amotosalen is already in use reducing pathogen activity in plasma [7].

Most of the above described methods require separation of the plasma from the employed chemicals, posing an additional effort and the need for testing of rest substance. Thus, ultrasound as a purely physical method poses a novel, effective and easily usable alternative.

Ultrasound used for sonication describes sound waves at a frequency above approximately 20 kHz, non-audible to humans, generated by electrically triggered quartz resonators and radiated in potent bundle format. The transmittable energy finds a high range of use in industry and military as well as in medical setups, e.g., to detach bacteria in clinical microbiological diagnostics or to detect implant-associated infections [8]. To inactivate all harmful microorganisms, especially viruses, one of the desired effects of ultrasound can be soft and hard cavitation [9], potentially causing mechanical stress or even damage [10]. Cell damaging effects of ultrasound rely on mechanical destruction of cell walls, change of conformation of proteins and carbohydrates and damage of free DNA through hard cavitation [11].

The goal of the underlying study is the detailed examination of ultrasound treatment for inactivation of transfusion-associated viruses including proof of virus inactivation by ultrasound, detection of optimal physical parameters such as time, frequency and power of ultrasound treatment and evaluation of ultrasound’s effects on the coagulation activity of plasma proteins. Ultrasound triggers chemical (radical) and mechanical (cavitation) reactions. In presence of oxygen, the radical reactions can destroy proteins, including coagulation factors [12]. Initial goal of the study was to find the ideal time window for least damage on coagulation factors in combination with maximum effect on virus inactivation. In the first part, specific effects of ultrasound under oxygen or shielding gas [13] on coagulation proteins were tested. The modifications were compared via measurement of partial thromboplastin time according to Quick (PT), activated partial thromboplastin time (aPTT), fibrinogen, coagulation factors such as FIX and FVIII to evaluate effects on extrinsic and intrinsic coagulation.

## Materials and methods

### Influence on coagulation by sonication

Initially, reduction of global tests such as Quick, elongation of aPTT and reduction of factors fibrinogen, FIX and FVIII after 60-min ultrasound treatment in varying frequency were tested and compared. Each test was repeated five times. Ultrasound baths of various sizes and sources for various frequencies from 27 kHz up to 250 kHz were employed, yielding a variation of power density, as described in Table 1.

**Table 1:** Employed ultrasound equipment and properties

Frequency (kHz)	Ultrasound power P_p_/P_RMS_ (W)	Bath size (mm^3^)	Numbers of PEZ elements	Area (cm^2^)	Power density (W/cm^2^)
27	1200/600	250 × 300 × 300	12	750	1.60
33	160/80	240 × 100 × 140	1	336	0.48
42	1200/600	250 × 300 × 300	12	750	1.60
70	400/200	300 × 200 × 250	4	750	0.53
250	160/80	240 × 100 × 140	1	336	0.48

*PEZ* piezo-electric element, *P*_*P*_ peak to peak value, *P*_*RMS*_ root mean square value

Virus inactivation was conducted in standardized 150 ml Compoflex^®^ bloodbags, the connecting tube shortened to 1 cm and the interface connected via a 3-way-connector. The bag was then stabilized by a holding frame inside the ultrasound container. For CO_2_ fumigation the blood bags were inserted into a custom-made pressure chamber.

Ultrasound treatment of standardized and documented virus suspensions in PBS and plasma as outlined in Table 5 was always conducted in the exact same procedure: 50 ml plasma per bag were treated, after the procedure samples were immediately frozen at − 80 °C until analysis. Ultrasound treatment under oxygen was performed at 27 kHz and 42 kHz at a power density of 1.60 W/cm^2^. Two blood bags were prepared, one as a control group, the other as the treatment test. Times of sampling (5 ml per sampling time) can be seen in Table 2:

**Table 2:** Sampling times in the presence of oxygen

Frequency (kHz)	Power density (W/cm^2^)	Ultrasound treatment period (min)	Taken samples with/without ultrasound (US) treatment	
			Positive control (without US)	Sonicated samples (with US)
27	1.60	60	0 min/60 min	60 min/every 10 min
42	1.60	60	0 min/60 min	60 min/every 10 min

**Fig. 1:**
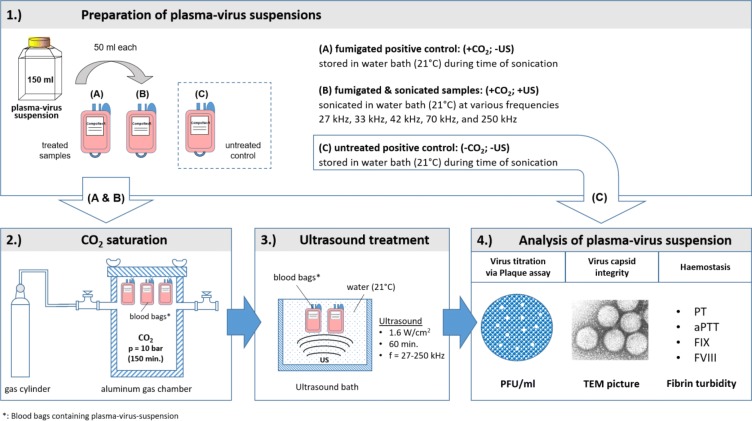
Preparation and sequential treatment of plasma–virus suspensions

For sequential testing of plasma, virus suspension and PBS, three bags were employed as shown in Fig. 1. One bag served as an untreated control group, the other two were saturated for 150 min at 10 bar with carbon dioxide, while one served as the CO_2_ control group, the other was used for ultrasound sampling.

The positive controls were kept for an equal time period in the water container under the exact same conditions. Acoustic exposure was then conducted for 5 h and samples taken in pre-defined time intervals. High power density tests were sampled every 30 min, low power every 60 min as described in Table 3.

**Table 3:** Parameter and timing of sampling dependent on frequency and power density of ultrasound treatment

Frequency (kHz)	Power density (W/cm^2^)	Sampling		
		Positive control 1, −CO_2_, −US	Positive control 2 +CO_2_, –US	Treated sample +CO_2_, +US
27	1.60	0 h/5 h	5 h/every 30 min	5 h/every 30 min
42	1.60	0 h/5 h	5 h/every 30 min	5 h/every 30 min
33	0.48	0 h/5 h	5 h/hourly	5 h/hourly
70	0.53	0 h/5 h	5 h/hourly	5 h/hourly
250	0.48	0 h/5 h	5 h/hourly	5 h/hourly

Fresh frozen plasma for the conduction of all tests was obtained from blood donation service Baden-Württemberg. Inactivation testing of virus loaded plasma was conducted at the Paul-Ehrlich-Institute as outlined below. Plasma was thawed at 37 °C, portioned in 50-ml units and processed immediately.

### Inactivation of viral test microorganisms

Inactivation of the model viruses was conducted in PBS. Sample viruses employed are listed in Table 4:

**Table 4:** Virus samples provided by the Paul-Ehrlich-Institute for experiments

Type of test virus	Family	Model for	Genome	Size (nm)	Cell line
PPV Porcine Parvovirus	Parvoviridae	B19	ssDNA	18–26	PK13
BVDV Bovine viral diarrhea virus	Flaviviridae	Hepatitis C	ssRNA	40–50	MDBK
SFV Semliki Forest Virus	Togaviridae	Hepatitis C	ssRNA	60–80	Vero
prv Pseudorabies virus	Herpesviridae	Herpes	dsDNA	150–200	ML

Properties and cell lines relevant for amplification and titration

To measure maximum CO_2_ saturation of plasma (for optimum removal of oxygen), partial pressures of CO_2_, O_2_, and pH values were tracked using the blood gas measurement system Synthesis 10 (Werfen GmbH, Vienna).

AdV-luc is a replication-deficient Adenovirus of human serotype 5 sized at roughly 90 nm with a double-stranded DNA. The so-called firefly luciferase gene is expressed under the control of the CMV promotor.

Biologic activity of coagulation proteins was conducted via measurement of the global thromboplastin time using the Quick test (PT), as well as the activated partial thromboplastin time (aPTT).

Virus count was conducted after staining with trypan blue to differentiate viability of cells. Cell count was conducted via microscope in a Neubauer counting chamber. Cytotoxicity was evaluated using the WST-1 assay.

Samples sonicated at 27 kHz under shielding gas protection were used for electrophoretic examination. Initially, the positive control samples (−CO_2_, −US and +CO_2_, +US) at the beginning (0 h) and end of sonication (5 h) were applied, followed by samples of plasma after 1, 2, 3, 4 and 5 h, separated in 8–16% Tris-Glycine-Gradient Gel at a voltage of 125 V and output of 57 mA. The recombined SDS-Page-Protein-Marker was used as a mass standard in the range of 10–150 kDa. Prior to electrophoretic analysis of the plasma samples, the albumin fraction was reduced, using an albumin removal kit by diluting 60 µl of plasma with 540 µl of binding buffer.

The extraction column was put onto a solid phase chamber, equilibrated with 1 ml of binding buffer and the diluted sample applied to the column. Further elution was conducted via gravity and the sample collected in a sample jar. The column was then flushed twice using 600 ml binding buffer. The collected extracts contained the albumin reduced sample.

For electrophoresis the sample was then in a ratio of 1:1 diluted with an extraction buffer. Additionally, one drop of Bromphenol blue was added to control the electrophoretic characteristics and one drop of sugar solution to load the sample. 6 µl of each sample were applied to the gel.

The virus titer was measured employing standard endpoint titration in eight replication cycles. The virus containing solution was diluted until no cytotoxic effect was measurable. Virus was sowed, diluted, titrated and in the end the tissue culture infective dose, TCID_50/ml_ was measured. The plates were examined regarding cytopathic effects caused by virus infections. Calculation was conducted in computer support based on probit analysis.

### Statistical analysis

During data evaluation mean values and standard deviations were calculated from at least five replicates. Calculation of mean values from several measurements was accompanied by the Gaussian error propagation law. The Student’s *t* test was conducted to get information about significances at a *p*-level of 0.05 by using Microsoft Excel 2016.

## Results

### Influence on coagulation by sonication

Virus inactivation in blood plasma can only be regarded successful if simultaneously proteins for coagulation remain more or less intact by choosing an optimum time window and energy level. Influence of sonication treatments at 27 and 42 kHz for 60 min on coagulation parameters (*n* = 5) are shown in Table 5:

**Table 5:** Mean reduction in percent of global tests such as Quick, elongation of aPTT and hemostasis factors as fibrinogen, FIX and FVIII after 60-min ultrasound treatment

	Reduction via sonication treatment	
Hemostasis parameter	27 kHz P/A = 1.60 W/cm^2^	42 kHz P/A = 1.60 W/cm^2^
Quick	67 (± 6) %	54 (± 5) %
aPTT	100 (± 9) %	111 (± 10) %
Fibrinogen	27 (± 2) %	32 (± 3) %
FIX	40 (± 3) %	40 (± 4) %
FVIII	78 (± 7) %	75 (± 7) %

**Fig. 2:**
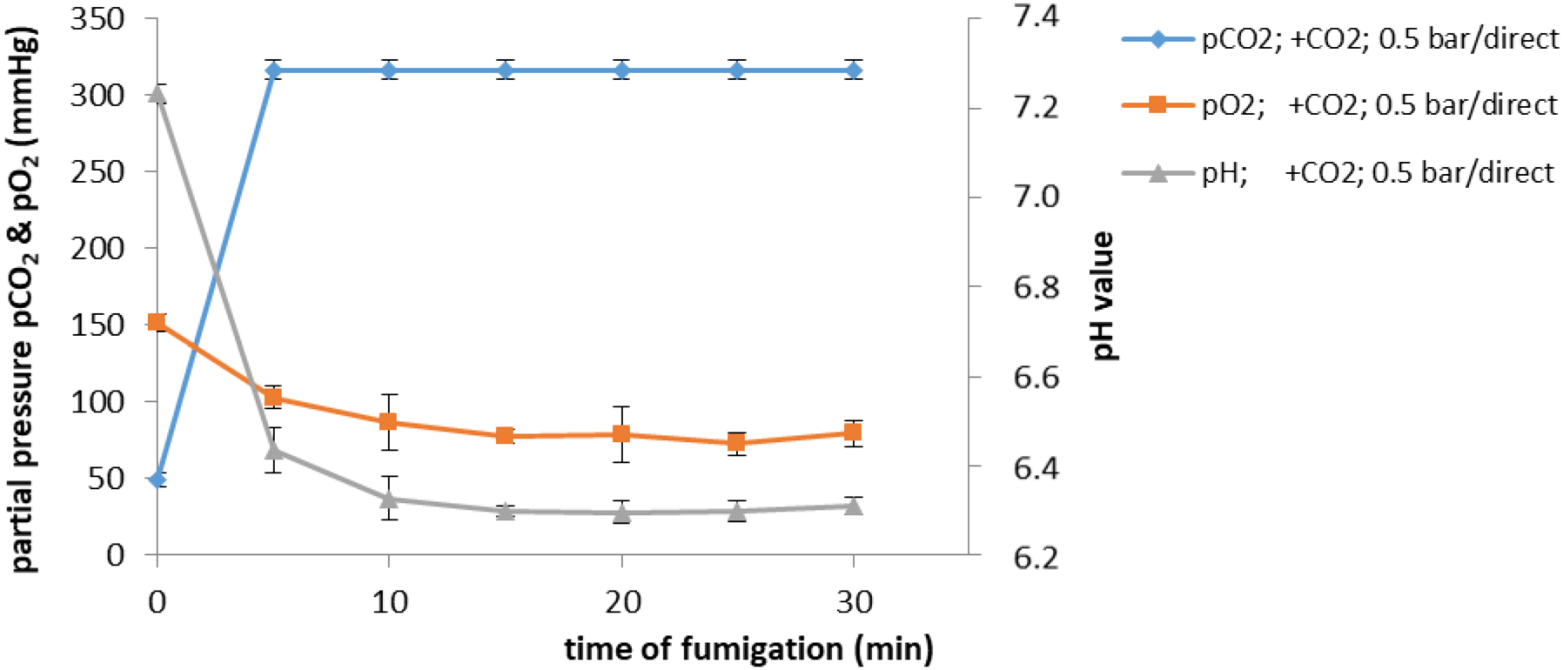
Influence of CO_2_ gas treatment on partial CO_2_ and O_2_ pressures as well as pH value (*n* = 6)

Preliminary literature and testing showed the protective function of CO_2_ insufflation prior to the ultrasound treatment. Displacement results of O_2_ by CO_2_ as a shielding gas by inserting carbon dioxide at 0.5 bar pressure can be seen in Fig. 2.

The used plasma samples showed an initial partial pressure of CO_2_ of 48 mmHg and pO_2_ of 151 mmHg. Introduction of CO_2_ led to a steep rise in pCO_2_ an equivalent linear decrease in pH from 7.3 to 6.3. In contrast, pO_2_ decreased relatively slowly.

Testing the activity of plasma coagulation factors after CO_2_ gas insufflation showed a marginal influence on overall clotting tests such as thromboplastin time and aPTT. On the other hand, activity of FVIII decreased by 10% and FIX by 18% on average.

Examination of the influence of ultrasound treatment on Quick, on aPTT, or coagulation factors such as fibrinogen, Factor IX and Factor VIII in summary showed the following: Extensive length of acoustic exposure yielded a reduction of coagulation activity in plasma, strongest influence displayed at low frequencies with high power density. Strongest influence was shown at 250 kHz on factor VIII activity, yielding a reduction of 68% (180 min) and 75% (300 min). Lowest influence was proven for a frequency of 70 kHz as shown in Table 6.

**Table 6:** Mean reduction effects on hemostasis parameters after sonication (*n* = 5) using additional CO_2_-shielding in relation to ultrasound frequency and duration (percentage reduction in comparison to untreated blood samples)

Sonication treatments using CO_2_-shielding (+CO_2_; +US) for hemostasis investigations										
Frequency (kHz)	27		42		33		70		250	
Power density (W/cm^2^)	1.60		1.60		0.48		0.53		0.48	
Duration (min)	180	300	180	300	180	300	180	300	180	300
Mean reduction of various hemostasis markers (%)										
Quick	10 (± 1)	24 (± 2)	9 (± 1)	24 (± 2)	5 (± 1)	7 (± 1)	2 (± 1)	3 (± 1)	9 (± 1)	17 (± 2)
aPTT	11 (± 1)	26 (± 3)	14 (± 2)	29 (± 3)	2 (± 1)	6 (± 1)	1 (± 1)	1 (± 1)	15 (± 1)	26 (± 2)
Fibrinogen	30 (± 2)	35 (± 4)	32 (± 3)	38 (± 2)	27 (± 1)	32 (± 2)	21 (± 2)	28 (± 2)	29 (± 3)	36 (± 2)
FIX	0 (± 0)	0 (± 0)	0 (± 0)	0 (± 0)	0 (± 0)	0 (± 0)	0 (± 0)	0 (± 0)	5 (± 3)	6 (± 3)
FVIII	51 (± 5)	61 (± 6)	48 (± 5)	53 (± 5)	52 (± 4)	55 (± 5)	28 (± 4)	33 (± 4)	68 (± 6)	75 (± 5)

Pre-testing for side effects has thus proven the frequency of 250 kHz to be unsuitable. For subsequent testing purposes both lipidated and non-lipidated viruses were examined. The observed viruses differ in size, structure of particles as well as structure of nucleic acid and cover the entire range of transfusion-associated viruses regarding characteristics as RNA-virus, DNA-virus, lipidated and non-lipidated viruses.

Results of influence on coagulation and virus count reduction under CO_2_ shielding gas protection are displayed in Tables 7 and 8.

**Table 7:** Mean reduction in % of coagulation activity under CO_2_-shielding gas at hard cavitation at a frequency of 27 kHz (*n* = 5)

Time	60 min	120 min	180 min	240 min	300 min
Reduction of coagulation activity under CO_2_-shielding gas (%)					
Quick	0 (± 0)	5 (± 2)	10 (± 2)	17 (± 2)	24 (± 2)
aPTT	0 (± 0)	5 (± 2)	11 (± 2)	18 (± 3)	26 (± 3)
Fibrinogen	11 (± 2)	23 (± 2)	30 (± 2)	33 (± 3)	35 (± 3)
FIX	0 (± 0)	0 (± 0)	0 (± 0)	0 (± 0)	0 (± 0)
FVIII	23 (± 4)	41 (± 5)	51 (± 5)	58 (± 5)	61 (± 5)

**Table 8:** Mean log_10_-reduction (± standard deviations) of virus titer under CO_2_-shielding gas via hard cavitation at a frequency of 27 kHz (*n* = 5)

Time	60 min	120 min	180 min	240 min	300 min
Mean log-reduction of virus titer in PBS (log_10_TCID_50_/ml)					
BVDV	1.7 (± 0.1)	3.8 (± 0.2)	≥ 3.8 (± 0.2)	≥ 3.8 (± 0.2)	≥ 3.8 (± 0.2)
SFV	0.7 (± 0.1)	2.7 (± 0.1)	4.2 (± 0.3)	5.0 (± 0.3)	≥ 5.7 (± 0.3)
PRV	1.0 (± 0.1)	3.0 (± 0.2)	4.7 (± 0.3)	4.5 (± 0.3)	≥ 5.5 (± 0.4)
PPV	0 (± 0)	0 (± 0)	0 (± 0)	0 (± 0)	0 (± 0)
Mean log-reduction of virus titer in plasma (log_10_TCID_50_/ml)					
BVDV	0.5 (± 0.1)	1.6 (± 0.1)	1.3 (± 0.1)	2.4 (± 0.2)	2.5 (± 0.2)
SFV	0 (± 0)	0 (± 0.1)	1.1 (± 0.1)	2.9 (± 0.2)	4.6 (± 0.3)
PRV	0.6 (± 0.1)	1.6 (± 0.1)	1.9 (± 0.1)	2.8 (± 0.2)	3.4 (± 0.3)
PPV	0 (± 0)	0 (± 0)	0 (± 0)	0 (± 0)	0 (± 0)

### Influence on coagulation under CO_2_-shielding gas

See Table 7.

### Inactivation of viral test microorganisms

See Tables 8.

## Discussion

Rising quantity of blood transfusions as well as a variety of infectious sources requires modern affordable and reliable methods for inactivation of viruses in blood while simultaneously protecting blood coagulation factors from negative influences. Success and failure of virus inactivation is measured in terms of multiple logarithmic reduction of virus titers. Influence of ultrasound on virus inactivation has been tested and described in literature yielding varying rates of success [14] partially describing the necessity for additional physical methods [15]. Other methodologies such as temperature changes as previously tested may prove generally efficient in virus inactivation, however, unsuitable for use on plasma products [16, 17].

Previous authors have tested similar methodologies, including pasteurization with regard to its effectiveness on virus inactivation and similar to our research proven the necessity of additional protective measures [18]. Others have tested the effectiveness of light waves on virus inactivation, but so far not yet with regard to human plasma products [19]. Photodynamic radiation has been tested as a method of virus inactivation, however frequently involving the risk of hard-to-control side effects such as undesired destruction or mutation of radiated material [20]. Inactivation of influenza A viruses (IAVs) via sterilization equipment with light-emitting diodes (LEDs) at peak wavelengths of 365 nm (UVA-LED), 310 nm (UVB-LED), and 280 nm (UVC-LED) was tested. These tests proved a decreased accumulation of intracellular total viral RNA in infected Madin–Darby canine kidney cells as well as a suppressed accumulation of intracellular mRNA (messenger RNA), vRNA (viral RNA), and cRNA (complementary RNA), as measured by strand-specific RT-PCR [21]. UVA light’s side effects on blood coagulation has undergone substantial testing in rabbit ear bleeding time. Tests showed that bleeding time influence of radiated human platelets is highly dependent on UVA dose indicating that virucidal AMT/1× UVA treatment does not influence platelet hemostatic function [22]. Furthermore, non-ionic surfactants such as Triton X-100 in combination with heat inactivation at 60 °C for 1 h have been tested in varying concentrations, however so far yielding sufficient effectiveness only for the herpes simplex virus, awaiting further results [23].

The method examined in our test series has proven to be effective in virus inactivation yet protective for blood’s hemostatic properties. The examined mechanical method yields satisfying results with regard to virus inactivation through hard cavitation as described earlier [24]. The effectiveness of the mechanical method as shown on the model viruses may be caused by a combination of various reasons, based on two hypotheses: Hypothesis one assumes that ultrasound destroys the lipid cover of the viruses or the capsid as proclaimed 75 years ago by Goldman and Lepeschkin [25]. Cavitational effects are assumed to mechanically fracture cells, as proven electron microscopically [26]. Hypothesis two assumes ultrasound to destroy the virus genome by mechanically breaking up sugar–phosphate bridges within the DNA via cavitation as well as destruction of hydrogen bridges via radical reactions [24, 27].

Other authors describe interaction of free radical species development during sonolysis [28]. Hard versus soft cavitational effects as described above were examined and their effectiveness studied [9]. Ultrasound treatment under protection of shielding gas seems to offer an appropriate method for microbiologic treatment of blood products as it is both affordable, safe and can be employed leaving the relevant blood components such as coagulation proteins unimpaired. The lack of addition or subsequent removal of chemical substances renders the procedure both safe and suitable for less developed countries.

Limitations of this study include the small selection of comparable virus stems compared. Yet these virus stems were chosen diligently to allow for a high degree of comparability. The examined method is both time and labor intensive as well as only functional for small amounts of treated blood cells. Consequently, the technology is hardly scalable for industry in developed regions. However, the described method allows a significant increase in safety of blood transfusions in technically less developed regions.

## Conclusion

It was clearly shown that ultrasound treatment yields positive results with regard to virus inactivation with minor side effects on coagulation proteins and no signs for cytotoxicity. In addition, hard and soft cavitation effects were compared which clearly showed that influences on virus inactivation and on coagulation factors occurred in different energy ranges. This allows for optimization of mechanical effects. In addition, the new method could potentially be combined with existing physical and chemical methods such as methylene blue or UV radiation.

## Outlook

Further studies may examine the effectiveness on a larger scale, industrialized model. In addition, cost and time consumption could be evaluated to understand the economic effects caused by this new technology.

## Data Availability

All relevant data are included in the manuscript. Further detailed data are available upon request.
